# Risk assessment to prevent heart failure

**DOI:** 10.18632/aging.102444

**Published:** 2019-11-14

**Authors:** Arjun Sinha, Donald M. Lloyd-Jones, Sadiya S. Khan

**Affiliations:** 1 Department of Preventive Medicine, Northwestern University Feinberg School of Medicine, Chicago, IL 60611, USA; 2 Division of Cardiology, Department of Medicine, Northwestern University Feinberg School of Medicine, Chicago, IL 60611, USA

**Keywords:** prevention, heart failure, aging

The rising prevalence of heart failure (HF) is a critical public health issue that requires urgent intervention. Age is a major risk factor for HF with preserved or reduced ejection fraction. Given the rising age of the population, the prevalence of HF will undoubtedly continue to rise. In the United States, HF is expected to affect more than 8 million people by 2030 with associated annual health care costs exceeding $70 billion [1]. Thus, aggressive prevention of HF in higher risk populations is imperative and consistent with national guideline recommendations by the American College of Cardiology, American Heart Association, and Heart Failure Society of America [2].

In order to better risk stratify asymptomatic individuals, Khan et al. developed a prediction model using pooled individual-level data from 5 diverse cohorts called the Pooled Cohort Equations to Prevent Heart Failure (PCP-HF) [3]. All cohorts were population-based samples with direct measurement of risk factors, adjudication of incident HF, and continued surveillance with at least 12 years of follow-up. The model was externally validated in two separate cohorts. The equation estimates the 10-year risk of incident HF in black and white men and women between the ages of 30 and 79 without baseline cardiovascular disease (CVD). The equation was developed from sex- and race-specific proportional hazards models that included: age, systolic blood pressure, antihypertensive medication use, body mass index, total cholesterol, high-density lipoprotein cholesterol, current smoking status, fasting glucose, diabetes medication use, and electrocardiogram measurement of QRS duration. External validation illustrated good discrimination (c-statistic ranging from 0.71 to 0.88) and good calibration (Greenwood Nam D’Agostino χ^2^<20) in black and white women and men.

The PCP-HF risk prediction model allows for quick and cost-effective screening to identify those high-risk individuals who require aggressive risk factor modification and may benefit from targeted therapies in order to prevent HF. Along with lifestyle changes, such approaches may include intensive blood pressure lowering, use of specific anti-hypertensive therapies (e.g. angiotensin receptor blockers) or novel diabetic therapies (e.g. sodium-glucose transport protein 2 inhibitors). While these treatment goals should lead to a decrease in incident HF, aging remains an important and to-date, non-modifiable risk factor. Therefore, understanding the pathophysiology of aging that contributes to development of HF will also be important. Identification of novel biomarkers that help differentiate pathologic from healthy aging may allow for more robust discrimination and risk stratification as well as may guide development of personalized and targeted therapeutic strategies in the future.

Future models should incorporate the concept of estimating and communicating lifetime risk of HF, since quantification of short-term risk alone provides incomplete risk stratification over the life course. Individuals at low predicted 10-year risk may in fact be at high lifetime risk of HF. This was shown for atherosclerotic CVD in data from the National Health and Nutrition Examination Surveys, where an estimated two-thirds of US adults with low short-term risk had high lifetime risk for atherosclerotic CVD [4]. Lifetime risk of developing HF to age 95 in middle-aged adults is high and ranges from 20-46% in black and white men and women [5]. Determining a risk threshold for intervention based on lifetime risk will be important in future studies as younger patients who have a high lifetime risk may be able to delay their initial event by multiple years (extend healthspan) as well as increase overall survival (extend lifespan) with early risk factor control, resulting in absolute and relative compression of morbidity.

Similarly, it will be important to consider comprehensive genetic screening for HF to identify younger individuals at increased lifetime risk of HF. This could be in the form of polygenic risk scores, as has been attempted for coronary artery diseae (CAD) [6]. However, polygenic risk scores for CAD have yet to demonstrate clinical utility, and therefore this approach alone may also not be ideal for HF. Identification of common variants that are differentially observed in race-specific groups and are related to cardiac mechanics as well as incidence of HF may offer an opportunity to identify unique genetic risk enhancers. Two examples of this include the V122I (valine-to-isoleuncine) mutation in the transthyretin (TTR) gene, which has a prevalence of 4% in blacks, and the H63D (histidine-to-aspartate) mutation in the major histocompatibility complex class I-like transmembrane protein (HFE) gene, which has a prevalence of 20% in whites. The V122I mutation is associated with adverse cardiac mechanics and increased risk of HF in elderly blacks while the H63D mutation is associated with development of hypertension, but whether it increases the risk of HF needs further investigation [7,8].

In summary, the PCP-HF model is unique and represents a generalizable tool for application in primary prevention populations due to inclusion of young and middle aged adults, representative sampling of the black community, no baseline CVD, and a lack of reliance on serum based biomarkers or cardiac imaging. While limitations include the inability to discriminate between HF with reduced or preserved ejection fraction, prevention strategies for both subytpes are largely similar. Lack of other racial groups limits broader application and warrants further investigation. Implementation of the proposed HF risk score will allow for broad initial and cost effective screening in the primary care setting and identification of next steps for additional screening and therapeutic management. The future of risk prediction in HF has arrived and prevention is key to mitigate the growing burden of disability and mortality related to HF.
